# The *Int7G24A *variant of transforming growth factor-beta receptor type I is a risk factor for colorectal cancer in the male Spanish population: a case-control study

**DOI:** 10.1186/1471-2407-9-406

**Published:** 2009-11-20

**Authors:** Adela Castillejo, Trinidad Mata-Balaguer, Carla Guarinos, María-Isabel Castillejo, Ana Martínez-Cantó, Víctor-Manuel Barberá, Paola Montenegro, Enrique Ochoa, Rafael Lázaro, Carmen Guillén-Ponce, Alfredo Carrato, José-Luís Soto

**Affiliations:** 1 Molecular Oncology Group, Elche University Hospital, Camino Almazara 11, 03203 Elche, Spain; 2 Genetic Counseling in Cancer Unit, Elche University Hospital, Camino Almazara 11, 03203 Elche, Spain; 3 Molecular Biopathology Department, Castellon Provincial Hospital. Avenida Doctor Clara 19, 12002 Castellon de La Plana, Spain; 4 Pathology Department, La Plana Hospital, Partida Carinyena Km 0.5, 12540 Vila-real, Spain

## Abstract

**Background:**

The *Int7G24A *variant of transforming growth factor-beta receptor type I *(TGFBR1) *has been shown to increase the risk for kidney, ovarian, bladder, lung and breast cancers. Its role in colorectal cancer (CRC) has not been established. The aims of this study were to assess the association of TGFBR1*Int7G24A variant with CRC occurrence, patient age, gender, tumour location and stage.

**Methods:**

We performed a case-control study with 504 cases of sporadic CRC; and 504 non-cancerous age, gender and ethnically matched controls. Genotyping analysis was performed using allelic discrimination assay by real time PCR.

**Results:**

The *Int7G24A *variant was associated with increased CRC incidence in an additive model of inheritance (*P *for trend = 0.005). No significant differences were found between *Int7G24A *genotypes and tumour location or stage. Interestingly, the association of the *Int7G24A *variant with CRC risk was significant in men (odds ratio 4.10 with 95% confidence intervals 1.41-11.85 for homozygous individuals; *P *for trend = 0.00023), but not in women. We also observed an increase in susceptibility to CRC for individuals aged less than 70 years.

**Conclusion:**

Our data suggest that the *Int7G24A *variant represents a risk factor for CRC in the male Spanish population.

## Background

Transforming growth factor beta (TGF-β) is one of the most potent inhibitors of proliferation in epithelial, neuronal and hematopoietic cells. The effects of an activated TGF-β signalling pathway include inhibition of cell cycle progression, promotion of terminal differentiation and activation of cell death [1].

TGF-β receptor type I (encoded by *TGFBR1*) is a mediator of TGF-β growth inhibitory signals. Extracellular TGF-β binds first to the type II receptor, which then dimerizes and activates the type I receptor, sending the signal to the nucleus via SMAD proteins [1]. Abnormalities of this signalling pathway are almost universal in cancer cells through a variety of mechanisms [2]. Among them are overexpression of the ligands, downregulation of receptors, and point mutations and deletions in the genes coding for proteins involved in the pathway [3]. Different *TGFBR1 *allelic variants have been associated with susceptibility to develop different types of tumours. Thus, the TGFBR1*6A allele, located at exon 1 (rs11466445) has been shown to be a susceptibility allele for colorectal cancer (CRC), breast and ovarian cancers, based on a meta-analysis of many case-control studies [4]. In contrast, one recent report found a lack of an association between this variant and CRC risk [5,6]. The role of this variant in CRC susceptibility remains controversial.

The *TGFBR1*_*Int7G24A *polymorphism (rs334354; NM_004612.2:c.1255+24G>A) has also been associated with cancer risk, but the function of this variant has yet to be discovered. Individuals carrying the *Int7G24A *variant have an increased risk of developing non-small cell lung cancer, renal cell carcinomas, transitional cell carcinomas of the bladder, and breast cancer [7-10]. To our knowledge, there is only one published report on the association between this genetic variant and CRC [11]. In that study, there was no evidence of an association between this polymorphism and colorectal cancer risk in familial cases.

To test whether the *Int7G24A *variant might be associated with the incidence of CRC in the Spanish population, we carried out a case-control study with 504 cases and 504 matched controls for age, gender and ethnicity. We found a significant association of the *Int7G24A *variant with the incidence of CRC. It also showed significant associations with patient gender (male) and age, but not with tumour location or stage. These data support the hypothesis that *Int7G24A *variant may be a low penetrance tumour susceptibility allele that predisposes to CRC mainly in men in the Spanish population.

## Methods

### Study subjects

A total of 504 sporadic cases of CRC and 504 controls from the Elche University Hospital and Castellon Provincial Hospital tissue banks were analysed in this study. Written consent was obtained from every patient to be included in the respective tissue banks. Patients diagnosed with a familial cancer syndrome were excluded. Thus, patients with Amsterdam II criteria for hereditary non-polyposis colorectal cancer were excluded, as were patients with Bethesda criteria and who were negative for microsatellite instability, who were studied in our genetic counselling unit.

This was a hospital-based case-control study. Controls were selected from the same hospitals with no personal history of cancer and selected according to diagnoses unrelated to the disease of interest. They were matched for age, gender and race/ethnicity with the study patients. The study was approved by the Ethics Committees from the Elche University and Castellon Provincial hospitals.

The median age for patients with CRC at diagnosis was 70 years (range 23-92); for controls it was 72 years (range 23-98). Gender distribution for the patients with CRC ('cases') was 59.4% men and 40.6% women; for controls, it was 53.3% and 46.7%, respectively. In 33.3% of the cases, tumours were in the proximal colon and 66.7% were located at the distal colon and rectum; 45% were classified as low stage (I and II) and 55% as high stage (III and IV). The numbers of cases and controls for whom information was unavailable for stratification were 64 and one, respectively.

### DNA and RNA isolation and cDNA synthesis

Control DNA samples were obtained from peripheral blood samples. DNA samples from cases were isolated after mechanical homogenization of non-cancerous frozen colorectal tissue. DNA isolation was performed using an EZ1 BioRobot (Qiagen, Valencia, CA, USA) according to the manufacturer's instructions. RNA was isolated from normal colon tissue of selected cases in the same manner. Synthesis of cDNA was performed using random primers and Reverse Transcription reagents (Applied Biosystems, Foster City, CA, USA).

### Genotyping the *Int7G24A *polymorphism (rs334354)

A total of 1008 individuals (504 cases and 504 controls) were analysed for this polymorphism. Single nucleotide polymorphism (SNP) analysis was performed using a real-time polymerase chain reaction (PCR) TaqMan assay for allelic discrimination (TaqMan SNP Genotyping Assay; ID: C_1413390_20; Applied Biosystems) with minor modifications. All PCR reactions contained 20 ng DNA, 10 μL TaqMan Universal Master Mix (Applied Biosystems), 0.5 μL of the assay and water for a final volume of 20 μL. The appropriate negative controls were also run in parallel. Real time PCR was performed on an ABI Prism 7300 Sequence Detection System (Applied Biosystems) using the following conditions: 95°C for 10 min and 40 cycles of amplification (95°C for 15 s and 60°C for 1 min). Software in the system was used to quantify the fluorescent signals from VIC- or FAM-labelled probes. Two representative cases for each genotype were sequenced directly to confirm the genotyping results. Sequencing reactions were performed using BigDye Terminator (version 3.1) Cycle Sequencing kits and run in an ABI Prism 3100-Avant Genetic Analyser (Applied Biosystems).

### Analysis of the lengths of Int7G24 and Int7A24 variant transcripts

Four cases previously genotyped as *G/G *and four genotyped as *A/A *were analysed to compare the length of their *TGFBR1 *transcripts. This experiment was performed using PCR and capillary electrophoresis. Primer sequences designated IVS7cDNA-F (labelled with 6-FAM) were 5'-AAATTGCTCGACGATGTTCC-3' for exon 7, and IVS7cDNA-R, 5'-CTCTGCCATCTGTTTGGGAT-3' for exon 8. The expected size of a normal PCR product was 151 bp. Twenty-five nanograms of DNA were used in a total PCR reaction volume of 25 μL AmpliTaq Gold PCR Master Mix 2 × (Applied Biosystems) and 20 pmol of each primer. After an initial 9 min at 95°C, PCR was run for 40 cycles of 95°C for 20 s; 55°C for 20 s and 72°C for 20 s; followed by 10 min final extension at 72°C. Diluted (1/10) PCR products along with Genescan 500 ROX size standards in HiDi formamide (Applied Biosystems,) were resolved by capillary electrophoresis (ABI 3100 Avant, Applied Biosystems) using POP6 polymer. GeneScan Software was used for the analysis of PCR fragment lengths (Applied Biosystems).

### Statistical analysis

We first examined the Hardy-Weinberg equilibrium of allelic distribution separately for cases and controls and then compared the allele frequencies between cases and controls. We evaluated the association of *Int7G24A *genotypes with CRC using multivariate unconditional logistic regression models assuming dominant (*A/A *and *G/A*, versus *G/G*), additive (*G/G *and *G/A*, versus *A/A*) or recessive (*A/A*, versus *G/G *and *G/A*) modes of inheritance, respectively. In our basic models, we adjusted for age and gender. We explored the potential effects of modification by age in an analysis stratified by the cases' median age at diagnosis (≤70 or >70 years). A χ^2 ^test was used to evaluate the differences in *Int7G24A *variant carrier frequencies between tumour and control groups for the dominant and the recessive models and to analyse any association between the SNP and clinical and pathological factors. Armitage's trend test was used to calculate *P *for trend in the additive model of inheritance. All *P *values were two-sided and *P *< 0.05 was considered significant. Results are expressed as the odds ratio (OR) and 95% confidence interval (CI).

## Results

### *Int7G24A *genotyping

**Figure 1:**
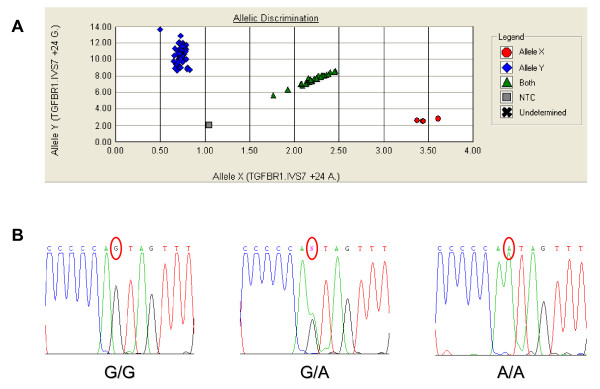
**Genotyping of the *Int7G24G>A *polymorphism**. (A) Real-time PCR for allelic discrimination. (B) Direct sequencing to confirm the genotypes.

Genotype distribution in the control and case populations did not deviate significantly from that expected for a population in Hardy-Weinberg equilibrium (*P *> 0.2). Allelic frequencies found in controls were 0.815 and 0.184 for the *Int7G24 *and *Int7A24 *alleles, respectively. For the patients with CRC, these frequencies were 0.764 and 0.236, respectively. Genotyping of the *Int7G24A *polymorphism is shown in Figure 1. The crude ORs assuming dominant, additive and recessive models of inheritance showed a significant increase of CRC risk for individuals carrying the *Int7G24A *variant. The best-fit model of inheritance was the additive one (*P *for trend = 0.005). Unconditional logistic regression adjusted for age, considering the median age for cases (70 y) as the threshold level, showed a statistically significant association of the *Int7G24A *variant with CRC susceptibility, for those individuals younger than 70 years (*P *for trend = 0.004). No statistically significant association was found between this polymorphism and individuals aged over 70 years (Table 1).

**Table 1:** Genotypes of the *Int7G24A *polymorphism in cases and controls and their association with CRC.

Int7G24A genotype	Cases (n = 504)	Controls (n = 504)	OR [95% CI]	*p*
Dominant model	number (%)	number (%)		
**G/G**	296 (58.73)	333 (66.07)	1 (ref)	
**G/A and A/A**	**208 (41.27)**	**171 (33.93)**	**1.37 [1.06-1.77]**	0.019*
Additive model				
**G/G**	296 (58.73)	333 (66.07)	1 (ref)	
**G/A**	178(35.32)	156 (30.95)	1.28 [0.98-1.67]	
**A/A**	**30 (5.95)**	**15 (2.98)**	**2.25 [1.20-4.23]**	0.005**
Recessive model				
**G/G and G/A**	474 (94.05)	489 (97.02)	1 (ref)	
**A/A**	30 (5.95)	15 (2.98)	**2.04 [1.11-3.85]**	0.032*
				
	≤ **70 yrs** **(n = 213)**	≤ **70 yrs** **(n = 219)**		
**G/G**, number (%)	118 (55.40)	150 (68.49)	1 (ref)	
**G/A**, number (%)	**80 (37.56)**	**61 (27.85)**	**1.67 [1.11-2.51]**	
**A/A**, number (%)	15 (7.04)	8 (3.65)	2.38 [1.00-5.68]	0.004**
				
	**>70 yrs** **(n = 227)**	**>70 yrs** **(n = 284)**		
**G/G**, number (%)	145 (63.88)	182 (64.08)	1 (ref)	
**G/A**, number (%)	72 (31.72)	95 (33.45)	0.95 [0.65-1.39]	
**A/A**, number (%)	10 (4.41)	7 (2.46)	1.79 [0.69-4.67]	0.662**
				
	**males** **(n = 295)**	**males** **(n = 226)**		
**G/G**, number (%)	166 (56.27)	160 (70.80)	1 (ref)	
**G/A**, number (%)	**112 (37.97)**	**62 (27.43)**	**1.74 [1.19-2.54]**	
**A/A**, number (%)	**17 (5.76)**	**4 (1.77)**	**4.10 [1.41-11.85]**	0.00023**
				
	**females** **(n = 202)**	**females** **(n = 277)**		
**G/G**, number (%)	126 (62.38)	172 (62.09)	1 (ref)	
**G/A**, number (%)	63 (31.19)	94 (33.94)	0.92 [0.62-1.36]	
**A/A**, number (%)	13 (6.44)	11 (3.97)	1.61 [0.71-3.65]	0.688**

* χ^2 ^test; ** *p *for trend (Armitage's trend test)

The results after gender adjustment were clear-cut, with no CRC risk association found for women and a strong statistically significant association for the presence of the *Int7G24A *variant and CRC in men (*P *for trend = 0.00023). The ORs for heterozygous and homozygous individuals for the *Int7G24A *variant were 1.74 (95% CI: 1.19-2.54) and 4.10 (95% CI: 1.41-11.85), respectively (Table 1). No significant associations were found between different genotypes and tumour location or stage.

### Lengths of *Int7G24 *and *Int7A24 *transcripts

We performed mRNA analysis from homozygous *G/G *and *A/A *individuals to test if the *Int7G24A *variant might be related to an abnormal mRNA splicing that could explain the function of this allele as a risk factor for CRC. However, the mRNA fragments obtained always had the expected size for normal mRNA splicing independently of the genotype.

## Discussion

The TGFβ pathway plays an important role in tumour development and progression [1]. Recently, there has been interest in the contribution of genetic variation in this pathway to cancer susceptibility. Several polymorphic variants in genes involved in this pathway have been studied [12]. The *TGFBR1_Int7G24A *variant has been previously associated with cancer risk, but the role of this variant has yet to be discovered. Zhang *et al *[7] reported that homozygous but not heterozygous carriers of this variant have an increased risk of developing non-small cell lung cancer. Chen *et al *[9] reported the association of this variant with the incidence of renal cell carcinomas (OR: 2.2; 95% CI: 1.22-3.96) and transitional cell carcinoma of the bladder (OR: 2.45; 95% CI: 1.8-3.16). In a meta-analysis of these three studies [8], the pooled OR was 1.76 (95% CI: 1.33-2.34; *P *< 0.0001). Another case-control study [10] found an association with breast cancer (OR: 2.42; 95% CI: 1.55-3.79), suggesting that the *Int7G24A *variant could also represent a risk factor for invasive and metastatic breast cancers (OR: 2.61; 95% CI: 1.65-4.11). Skoglund-Lundin et al. [11] did not detect a significant association between CRC risk and the *Int7G24A *variant when they analysed data from cases with a familial history of colorectal cancer. The expectedly low-penetrance effect of this putative susceptibility allele would be very difficult to detect in cases with high-penetrance alleles. Moreover, the authors did not stratify their data according to sex.

An exclusion criterion for the present study was the diagnosis of any familial cancer syndrome predisposing to CRC, such as hereditary non-polyposis colorectal cancer, familial adenomatous polyposis, Cowden disease and Peutz-Jeghers syndrome. We were interested to evaluate the effect of this SNP for sporadic CRC susceptibility and tumour progression. The potential effect of the SNP on familial syndromes as modifier of the penetrance, or the phenotype expression should be approached independently in further specific studies.

Control individuals were selected from those patients treated at an emergency department with diverse non-cancerous pathologies and with no personal history of cancer. The probability that controls suffered from diseases putatively caused by the same risk factors as the patients with CRC is extremely low.

It is well known that one of the main limitations and sources of confounding the results in association studies comes from population stratification and inappropriate sample sizes. *A priori*, our study with 504 sporadic cases and 504 controls allowed us to detect an OR of 1.5 for *G/A *heterozygous individuals (considering a frequency of 0.3) and an OR of 2.0 for *A/A *homozygous individuals (considering a frequency of 0.025) with 80% power (two-sided test, alpha level 5%). Estimated genotype frequencies were obtained as preliminary results in a subset of our control population and were similar to the frequencies registered at the SNP database for European populations http://www.ncbi.nlm.nih.gov/projects/SNP/. The results presented here show an increase of CRC susceptibility for those individuals carrying the *Int7G24A *variant allele with a dosage effect (additive model of inheritance). Adjusting the OR by age, we only observed an association of *Int7G24A *variant carriers with CRC risk in subjects aged less than 70 years (Table 1). Thus, the genetic risk effect is more evident in younger individuals. Ageing implies a huge amount of acquired predisposing carcinogenic factors that could overshadow the mild effect of the susceptibility alleles.

When we analysed the data adjusting by gender, we found that the *Int7G24A *variant was strongly associated with CRC risk in men but not women. We do not know why. Evidence is accumulating supporting gender-related differences in the development of CRC, but to our knowledge, few reports have been published regarding sex-specific relationship between SNPs and CRC. Slattery *et al *[13] reported that the oestrogen receptor beta CA repeat polymorphism was associated with an increase relative risk of colon cancer in women but not in men. On the contrary, increasing number of CAG repeats in the androgen receptor was directly associated with colon cancer among men, but not in women. Both oestrogen and androgen receptors are present in colorectal tissue and it has been suggested that these might be important in regulating the CRC risk associated with these hormones. Activation of the TGFβ signal transduction system is also subject to hormonal regulation [14]. This striking connection might suggest the involvement of sex steroid hormones acting through the TGFβ pathway in the aetiology of CRC.

The apolipoprotein E (ApoE) epsilon 2/3 polymorphism is another example of gender-specific modulation of CRC risk and prognosis where men have a highly significant association but there is no association in women [15]. Bae *et al *[16] found a gender-specific association between polymorphisms of the gene for vascular endothelial growth factor (*VEGF 936 C>T*) and CRC in the Korean population. T allele-bearing genotypes significantly increased the risk for CRC in women but not in men. Moreover, there are gender-related survival differences in association with *EGFR *(gene for epidermal growth factor receptor) polymorphisms in patients with metastatic colon cancers [17]. Again, oestrogens and androgens may be behind all these associations because of their functional links with ApoE [18], VEGF [19] and EGFR [20].

It is possible that the positive associations shown here were not based on real effects of this polymorphism, but rather reflect unknown differences in population ancestry between the case and control groups [21]. We consider the probability of false-positive inference attributable to population stratification to be small, because the cases and control individuals were recruited from an ethnically homogeneous population with no indication of a significant amount of recent genetic admixture.

Given our results and those in the literature, it seems reasonable to speculate that there might be a gender-dependent allelic architecture for CRC risk associated with sex steroids. This would introduce a new level of complexity in the 'common disease-common variant' hypothesis [22]. Our association studies between this polymorphism and clinicopathological factors did not show any statistically significant results. The lack of difference in genotype distribution between male and female patients with CRC might be attributed to the contribution and compensation of other gender-related susceptibility polymorphisms, where the risks affect women preferentially. To date, how this intronic SNP affects oncogenesis remains to be elucidated. It has been proposed that this SNP creates an alternative splice site within intron 7. *In vitro *analysis with breast and ovarian cell lines from carriers of the *Int7G24A *variant have shown the retention of the seventh intron in the mRNA up to the site of the *Int7G24A *variant [23]. We wanted to study if this alternative splicing also occurred in the normal colorectal tissue from patients with CRC. However, we concluded that factors other than alternative splicing might account for the association of this polymorphism with cancer, although we cannot rule out the possibility that *Int7G24A *variant is a marker representing a *TGFBR1 *haplotype. In this regard, it is interesting to note the likely 'bystander susceptibility effect' for the TGFBR1*6A allele because of linkage disequilibrium with the unknown causative mutation of the allele-specific expression (ASE) in the *TGFBR1 *gene [24]. In our series, no linkage disequilibrium was found between the TGFBR1*6A and Int7G24A variant (unpublished results). No information is available regarding the association between variant and ASE. Further studies are required to characterize the molecular mechanisms by which variant is involved in gender-specific susceptibility to CRC.

## Conclusion

The results of the present study strongly suggest that the *TGFBR1_Int7G24A *allele does confer an increased risk of colorectal cancer in the male Spanish population. The growing list of gender-dependent association of allelic variants and the risk of colorectal cancer might suggest the involvement of sex steroid hormones in the aetiology of CRC.

## Competing interests

The authors declare that they have no competing interests.

## Authors' contributions

AC participated in its design and coordination, in the molecular genetic studies and helped to draft the manuscript. TM-B, CG, M-IC, AM-C, V-MB participated in the molecular genetic studies and helped to draft the manuscript. PM participated in the design of the study, performed the statistical analysis and helped to draft the manuscript. EO, RL participated in samples biobanking and helped to draft the manuscript. CG-P, AC participated in the design of the study and performed the statistical analysis. J-LS conceived of the study, and participated in its design and coordination and drafted the manuscript. All authors read and approved the final manuscript.

## Pre-publication history

The pre-publication history for this paper can be accessed here:

http://www.biomedcentral.com/1471-2407/9/406/prepub
